# Supplementary material for: MetaRibo-Seq measures translation in microbiomes
Source: Nat Commun. 2020 Jun 29;11:3268. doi: 10.1038/s41467-020-17081-z (PMC7324362; doi:10.1038/s41467-020-17081-z)
Supplement: Supplementary file 10 — Supplementary Data 7 [file 41467_2020_17081_MOESM10_ESM.zip › File2/Confidence_High_Taxonomy/161681_out.krona.html]

Javascript must be enabled to view this page.

members
magnitude
magnitudeUnassigned
count
unassigned
taxon
rank

161681\_out

2

superkingdom
2
2

2
1239
phylum

class
186801
2

order
186802
2

family
216572
2

genus
1
2
459786

SRS142781\_contig\_number\_9601

1
species

SRS018575\_contig\_number\_545
876091
